# Anti-Libidinal Interventions in Sex Offenders: Medical or Correctional?

**DOI:** 10.1093/medlaw/fww003

**Published:** 2017-02-02

**Authors:** Lisa Forsberg, Thomas Douglas

**Affiliations:** 1 Centre of Medical Law and Ethics, King's College London, Strand, London WC2R 2LS, UK; 2 Uehiro Centre for Practical Ethics, Faculty of Philosophy, University of Oxford, Oxford, UK

**Keywords:** Chemical castration, Consent, Crime prevention, Criminal justice ethics, Medical ethics, Neurointerventions, Neuroethics

## Abstract

Sex offenders are sometimes offered or required to undergo pharmacological interventions intended to diminish their sex drive (*anti-libidinal interventions* or *ALIs*). In this paper, we argue that much of the debate regarding the moral permissibility of ALIs has been founded on an inaccurate assumption regarding their intended purpose—namely, that ALIs are intended solely to realise medical purposes, not correctional goals. This assumption has made it plausible to assert that ALIs may only permissibly be administered to offenders with their valid consent, in line with the approach taken to most other interventions with a medical aim. However, we argue that, contrary to this assumption, the state's intention in relation to at least some ALIs is, at least in part, to achieve correctional objectives. We evaluate two legal regimes for ALI provision—section 645 of the California Penal Code and the mental health regime in England and Wales. In each case, we identify the state's implicit purpose in imposing ALIs and argue that the Californian and English regimes both serve as counterexamples to the view that ALIs are intended solely for medical purposes. While the moral implications of our argument are not straightforward, it raises the question whether consent is required for permissible imposition of ALIs, and more generally, whether the moral permissibility of crime-preventing interventions using medical means should be assessed against the standards of medical ethics or against those of criminal justice ethics.

Sex offenders are sometimes offered, or required to undergo, pharmacological interventions intended to diminish their sex drive. The most well known of these interventions consists in the injection of hormone modulators that suppress testosterone activity to pre-pubescent levels, an intervention commonly referred to as ‘chemical castration’.^1^ Less well known is that some widely used anti-depressants, which can lower libido as a side effect when used to treat depression, have been used in sex offenders *for* these anti-libidinal effects.^2^ We will henceforth refer to pharmacological interventions administered for the purposes of reducing sex drive as *anti-libidinal interventions* or ALIs.

There is some evidence that, in certain classes of sex offenders,^3^ ALIs can be effective both in reducing the risk of recidivism and in mitigating the symptoms of paraphilias.^4^ Some forensic psychiatrists^5^ and politicians^6^ have called for greater use of ALIs in sex offenders, and some jurisdictions have recently effected legislative changes intended to provide for this.^7^ Nevertheless, the administration of ALIs to sex offenders has remained controversial, particularly when the ALI is imposed by the institutions of criminal justice, rather than being offered within a clinical psychiatric setting.^8^

This controversy has taken on an increased importance in recent years, as progress in the neuroscience and psychology of (anti-)social behaviour is beginning to suggest other possible means of mitigating risk factors for criminal conduct through pharmacological intervention. For example, selective serotonin re-uptake inhibitors, widely used to treat depression and anxiety disorders, have recently shown promise in reducing aggression.^9^ Similarly, the drug divalproex has been found to reduce impulsiveness in adolescents with explosive temper.^10^ It is not difficult to imagine that interventions such as these might ultimately be used, or advocated for use, in some violent offenders.

Unsurprisingly, then, ALIs have come to be seen as a test case for arguments that will have much wider applicability.^11^ In the future, the courts, and forensic psychiatrists, may have at their disposal a range of pharmacological interventions capable of addressing putative risk factors for criminal offending. Debate concerning the ethics of using ALIs in sex offenders may thus ultimately have wide implications for the use of pharmacological interventions in criminal offenders, and indeed some authors have already begun to widen the ethical discussion of ALIs so as to include a broader range of interventions.^12^

Given these potentially broad implications, it is important that debate on ALIs is grounded on accurate assumptions. In this paper, we aim to show that this has not been the case to date. More specifically, we argue that the debate has been founded on an inaccurate assumption regarding the intended purpose of ALIs.

## I. THE ASSUMPTION

Debate concerning the use of ALIs in sex offenders has focussed on their use as an optional alternative to (further) incarceration. It has most frequently addressed the practice of offering sex offenders early release from prison on the condition that they agree to undergo an ALI, not the (less common) practice of unconditionally requiring that an offender undergo an ALI. On the ‘early release’ model, the offender retains the option of avoiding the ALI, though doing so will result in further incarceration.

The dominant objection to the use of ALIs in this way has been that it is coercive and thus precludes the possibility of obtaining the offender's valid consent to the intervention. For instance, Kari Vanderzyl argues, in relation to chemical and surgical castration, that

the doctrine of informed consent requires a knowledgeable and voluntary decision to undergo treatment, yet offering a convicted offender castration as an alternative to a lengthy prison sentence constitutes an inherently coercive practice rendering truly voluntary consent impossible. Thus, castration should be rejected as a condition of probation.^13^

Similarly, William Green argues that

Voluntary consent depends upon a person's ability to make a choice freely. … The convicted rapist is faced with two options—a lengthy prison sentence or even death on the one hand and Depo-Provera [a form of chemical castration] or surgical castration on the other—and cannot be said to have the capacity to act freely in making a choice. Freedom of choice is impossible because the convict's loss of liberty constitutes a deprivation of such a magnitude that he cannot choose freely and voluntarily, but he is forced to give consent to an alternative he would not otherwise have chosen. In such circumstances men are willing to ‘barter their bodies.’ … As a consequence, the convicted rapist cannot give voluntary consent to an offer of probation which contains a surgical castration or Depo-Provera condition.^14^

The primary response to these claims has been to maintain that, though offenders offered a choice between undergoing ALIs and remaining incarcerated clearly face a strong incentive to consent to castration, that incentive does not render their consent invalid, for example because it does not amount to coercion, or if it does, it does not undermine autonomy.^15^

Notably, most commentators on both sides in this debate have accepted, at least implicitly, a consent requirement in relation to ALIs; they have accepted that valid consent is *required* for the morally permissible administration of ALIs, including to criminal offenders.^16^ (It is perhaps for this reason that they have not entertained the possibility that it might be permissible to impose ALIs on sex offenders without offering any alternative.) This common acceptance of the consent requirement betrays, we believe, an assumption that ALIs are intended to realise medical rather than correctional objectives. For if ALIs were intended wholly or in part to realise correctional objectives, the requirement for consent would be at the very least open to question. It is not normally taken to be a persuasive objection to traditional correctional interventions, such as incarceration and probation regimens, that they are imposed without the offenders’ consent. Indeed, even when pharmaceutical interventions have been used in ways that are clearly intended to realise correctional objectives—as in the case of the lethal injection, which has been used to execute death sentences—objections have not typically adverted to the lack of offender's consent. It is assumed to be permissible to perform highly intrusive and harmful interventions on criminal offenders without consent provided those interventions are intended at least in part for correctional purposes and are not susceptible to non-consent-based objections. Thus, if ALIs were conceived as intended partly or wholly for correctional purposes by those who have debated the ethics of their use, it would be surprising that these authors have typically accepted the requirement for consent, and even more surprising that they have typically accepted it without argument.^17^ On the other hand, we can easily account for acceptance of the consent requirement if we suppose that those who have accepted it have assumed that ALIs are being used solely for medical purposes.^18^ It is typically thought that, except in certain narrowly defined circumstances, interventions intended solely to achieve medical objectives ought to be administered to a competent person only with the consent of that person.^19^

This paper argues that, contrary to this assumption, some ALIs are intended, at least in part, to realise correctional objectives. We begin, in Section II, by offering some thoughts on how examining the legal regimes under which ALIs are provided might reveal the state's intentions in imposing ALIs. Using the framework developed there, we then proceed to analyse the purpose for which ALIs are provided in two jurisdictions: California, and England and Wales. California is examined in Section III, and England and Wales in Section IV. We present these two jurisdictions as counterexamples to the view that ALIs are intended solely for medical purposes, and we have chosen these jurisdictions because they exemplify two rather different ways in which ALIs may be used for correctional purposes. Section V draws out some of the possible moral implications of the argument and relates these to an argument offered recently by Jesper Ryberg.^20^

The analysis is not intended to be a comparative legal analysis of the two jurisdictions we discuss. Rather, we seek to illustrate how two different regulatory approaches to the provision of ALIs to sex offenders both involve aims that are correctional at least in part. The argument, then, is that if ALIs are at least sometimes intended for correctional purposes, this raises the possibility that the consent requirement is inapposite, morally speaking. The argument is not, however, that the consent requirement is certainly inappropriate, only that, where ALIs are intended for correctional purposes, the consent requirement cannot be assumed without argument (as it has been).

## II. MEDICAL AND CORRECTIONAL OBJECTIVES

There is little agreement on the purposes of medicine, and even less on the purposes of criminal justice. However, even if the two sets of objectives are understood inclusively, they arguably remain distinct. For the purposes of this article, an intervention will be understood to have a medical purpose when it is intended to (a) augment the recipient's wellbeing by (b.i) curing or mitigating a physical or medical disease or disorder, or (b.ii) modulating some other aspect of the recipient's biological functioning. An intervention will be understood to have a correctional purpose when it is intended to (a) mete out deserved suffering on the recipient (retribution), or (b) protect the public from crime by (i) preventing the recipient of the intervention from offending (for example, through incapacitation, rehabilitation, or specific deterrence), or (ii) deterring others from offending (general deterrence).

Note that, on this account, an intervention can have a correctional purpose without being *punitive*, in the sense that it is intended to mete out hardship or suffering. Psychological rehabilitation programmes, for instance, are typically non-punitive, but when intended to protect the public they would, on our view, nevertheless qualify as correctional in purpose.

Note also that a single intervention could be intended to realise both medical and correctional objectives; the categories are thus not mutually exclusive. For example, an intervention might be intended to *both* augment the recipients’ wellbeing *and* diminish his likelihood of re-offending *by* mitigating some disorder that is both an impediment to wellbeing and a risk factor for future offending. Many existing psychological rehabilitation programmes used in, for instance, substance users and sex offenders appear to have precisely this set of purposes. They aim to treat a substance abuse disorder or paraphilia in order both to prevent further offending and convey a direct health benefit to the offender.

It is, of course, not always straightforward to identify the state's purposes in providing an intervention. We will use four considerations to identify the intended purpose of an ALI: the institutional framework under which it is provided, the conditions that must be met in order for it to be provided, the provisions for the termination of the intervention, and the language in which the intervention is described. We submit that the following would count in favour of ascribing a medical purpose to an ALI:

- The ALI is provided at the direction of healthcare institutions (eg hospitals, clinics) under the oversight of a jurisdiction's department of health or its equivalent.
- The ALI is provided only on the condition that the recipient qualifies for a medical or psychiatric diagnosis which the ALI is likely to mitigate, and that the benefits of the intervention for the recipient are likely to outweigh the risks to him.
- If a repeated or temporally extended intervention, the ALI will be stopped when it is no longer of benefit to the recipient, or when he no longer suffers from the disorder or disease.
- The ALI is typically described in official materials using medical language such as ‘treatment’, ‘therapy’, and ‘cure’.

By contrast, the following would count in favour of ascribing a correctional purpose to an ALI:

- The ALI is provided at the direction of correctional authorities (eg prison services, parole boards, criminal courts, or probation centres) under the oversight of a jurisdiction's department of justice or its equivalent.
- The intervention is provided only on the condition that the recipient has committed or is likely to commit a crime (or a crime of a specified kind) and is provided irrespective of whether the recipient qualifies for a medical or psychiatric diagnosis.
- If a repeated or temporally extended intervention, the ALI will be stopped after a fixed and predetermined time has elapsed or on the individual's death (consistent with a retributive or deterrent objective) or when the offender's risk of re-offending falls below a given level (consistent with an incapacitative or rehabilitative objective).^21^
- The ALI is typically described using correctional language such as ‘sanction’, ‘punishment’, and ‘corrections’.

In what follows, we will appeal to these four dimensions to argue that ALIs are, in at least two jurisdictions, used in part for correctional purposes.^22^

It is important to stress again that this is not a comparative legal analysis; the aim is not to compare the statute providing for the use of ALIs in sex offenders in California to (one of) the regimes they could be provided under in England and Wales. Nor is it intended as an exhaustive review of use of ALIs for correctional purposes: ALIs have been used for such purposes in other jurisdictions as well. Rather, the aim is to present two legal frameworks under which ALIs can be provided, as counterexamples to the assumption that ALIs are intended solely for medical purposes. The particular jurisdictions have been chosen because they illustrate two rather different kinds of legal arrangement under which ALIs may be provided, both of which, we argue, represent correctional aims, at least in part.

## III. CALIFORNIA

This section aims to determine whether ALIs provided under California's specific ‘ALI statute’—section 645 of the California Penal Code—are intended for medical purposes, correctional purposes, or both.^23^ Section 645 allows for medroxyprogesterone acetate (an ALI often referred to by its trade name, Depo-Provera) or its chemical equivalent to be imposed on offenders as part of a criminal sentence.^24^ We argue that the use of ALIs under section 645 belies a correctional intent, although the precise nature of the correctional purpose is unclear and seems to involve a mixture of retribution and general deterrence.

### A. Institutional Setting

One factor suggesting that the provision of ALIs under section 645 is intended for correctional rather than medical purposes is its placement in the penal code.^25^ A further indication is that ALIs are provided at the direction of correctional authorities. *Courts* sentence individuals who have been convicted of certain sex offences to undergoing ALIs.^26^ This is at the court's own discretion after a first commission of the any of the relevant sexual offences,^27^ and as a matter of statutory requirement upon a second such offence.^28^ The statute requires that ALIs commence 1 week prior to the offender's release on parole and continue ‘until the Department of Corrections demonstrates to the Board of Prison Terms that this treatment is no longer necessary’.^29^ The fact that decisions about whether ALI provision continues to be necessary are made by correctional authorities is further suggestive of a correctional objective.

The statute does not contain any requirement for healthcare professionals to be involved, either in decisions about whether particular offenders should undergo ALIs, or in a supervisory capacity during the administration of the intervention, or in decisions about cessation of the ALI.^30^ This again seems indicative of correctional intent.

### B. Conditions of Provision

While legal instruments providing for the use of ALIs are not always clear about the aim of these interventions, one way to ascertain an implicit aim is to consider the criteria used to determine whether ALIs might be provided to particular offenders. In California, there are a number of factors that indicate that the use of ALIs under the statute is not intended to serve medical purposes. For example, neither section 645 (a) nor (b) states that ALIs should only be provided to offenders who are likely to benefit from them, nor do they require that offenders are screened for clinical suitability prior to being ordered to undergo ALIs, and there is no requirement that the offender qualify for a medical or psychiatric diagnosis.^31^ Section 645 applies to individuals who have committed certain sex offences against victims aged 13 years or under, and might therefore track paedophilia, which has been identified as one of the conditions for which ALIs may be clinically indicated.^32^ However, not everyone who commits a sex offence against a victim under the age of 13 years will suffer from paedophilia, or any other kind of paraphilia.^33^ As Edward A. Fitzgerald notes, ‘a diagnosis [of paraphilia] cannot be based solely on the offender's sexual behavior because such behavior may be motivated by a number of different causes’.^34^ Section 645 therefore allows for ALIs to be provided to offenders who do not qualify for a relevant diagnosis and who are therefore unlikely to benefit from them.^35^ This suggests that ALIs are not intended for medical purposes.^36^

A further indication that ALIs are not intended to realise medical objectives is that section 645(d) states that ‘[t]he parolee shall begin medroxyprogesterone acetate treatment one week prior to his … release from confinement in the state prison or other institution’.^37^ If ALIs were intended to treat a condition that is present throughout the period of incarceration, it is unclear why their provision would be delayed until this point. Kris Druhm argues that the fact that ALIs begin when the sentence is coming to an end suggests that ‘it is not part of the punishment but a separate treatment to help the subject upon release’.^38^ However, this claim appears to be undermined by the lack of screening for suitability to undergo the intervention, and the fact that ALIs are initiated too late for them to be likely to have an effect by the time of release.^39^

Section 645 also does not provide for ALIs to be used in conjunction with any form of counselling, psychotherapy, or other behavioural intervention (such as cognitive-behavioural psychotherapy) that would have been in line with the empirical evidence regarding their therapeutic effectiveness,^40^ and which is also the standard approach in many other jurisdictions where ALIs are used.^41^ Again, this suggests that the intent is not therapeutic.

Other commentators have noted that section 645 seems to provide for ALI use with a correctional rather than a therapeutic intent. For example, Kay-Frances Brody submits that the failure of section 645 to require ALIs to be tailored to individual offenders’ particular needs, along with the fact that the statute does not make reference to other treatment modalities ‘indicates quite forcefully that this provision is strictly punitive, rather than therapeutic’.^42^ Similarly, Raymond A. Lombardo contends that ‘[i]f California desired that these offenders receive treatment, the state would have followed the Depo-Provera research protocols, and limited the Depo-Provera application to paraphiliac offenders engaged in a counseling program’.^43^ On his view, ‘[t]hat the state disregarded the elements of the experimental treatment programs […] suggests that the state is not interested in treating offenders, but rather in punishing them’.^44^

There is, moreover, some evidence that ALIs provided under section 645 are intended specifically to achieve retributive or general deterrent objectives rather than preventing the recipient from re-offending (for example via rehabilitation, incapacitation, or specific deterrence). While the statute distinguishes between first time offenders and reoffenders, which to some extent may track likelihood of further reoffending, it fails to take into account other factors which have been found to predict risk of recidivism, and which are generally understood to be crucial in determining what kinds of interventions would be suitable, such as the offender's age, the motivation behind the offence, the presence of deviant sexual fantasies (paraphilic motivations), and antisocial orientation (nonparaphilic motivations).^45^ Considerations that determine offenders’ eligibility for undergoing ALIs under section 645 are backward looking (focussing on characteristics of the victim of the offence) rather than forward-looking (focussing on whether ALIs are likely to be effective in preventing recidivism or mitigating an underlying disorder). Although victim characteristics may track effectiveness in preventing recidivism to some degree (for example, to the extent that they track paraphilia), blanket use of ALIs on all offenders who commit certain sexual offences will undoubtedly result in ALIs being administered to some offenders for whom they will not reduce the risk of recidivism.^46^ This might suggest that ALIs provided under section 645 are not merely intended for correctional purposes, but specifically for retributive or general deterrent purposes, rather than for the prevention of recidivism in the offender subjected to the intervention.

### C. Conditions of Termination

A distinctive feature of interventions intended for medical purposes is that they are not legally time-limited. Rather, such interventions continue until the patient no longer qualifies for the relevant diagnosis or they no longer benefit the patient. By contrast, a common (though not universal) feature of interventions intended for correctional purposes is that they *are* time-limited; they cease once the offender has ‘served his time’.

In this respect, ALIs administered under section 645 might seem to conform to the medical paradigm; they are commenced 1 week prior to the offender's release on parole, and continued ‘until the Department of Corrections demonstrates to the Board of Prison Terms that this treatment is no longer necessary’.^47^ However, as we noted above, the fact that decisions about whether ALIs should cease or continue are made by correctional authorities, rather than healthcare institutions or healthcare professionals, seems to indicate that the purpose is not, in fact, medical. The statute does not set out the criteria used for determining whether ALIs continue to be necessary,^48^ but the fact that it does not contain any reference to medical criteria, nor require the involvement of healthcare professionals,^49^ suggests that ‘no longer necessary’ should not be interpreted as referring to an assessment of offenders’ clinical needs.^50^ This, again, seems to cast doubt on the suggestion that ALIs are provided for a medical purpose.

### D. Language

Section 645 does not contain straightforwardly correctional language, such as explicit references to ‘sanction’, ‘punishment’, or ‘correction’. Indeed, Druhm submits that ‘[i]t is possible to support the argument that §645 imposes treatment rather than punishment by an analysis of its actual wording’.^51^ He notes that section 645 states that ‘[t]he parolee shall begin medroxyprogesterone acetate *treatment* one week prior to his or her release from confinement … and shall continue *treatments* until the Department of Corrections demonstrates … that this *treatment* is no longer necessary’ (Druhm's emphasis),^52^ and contends that ‘[b]y labeling the Depo-Provera injections as treatments and providing that they will continue until no longer necessary, the statute suggests it is in fact trying to treat the subject rather than punish him’.^53^ Druhm also observes, however, that ‘[c]onversely, certain language in § 645 can be interpreted as suggesting the purpose of the castration is punitive in nature’.^54^ He notes that section 645(b) states that sex offenders convicted for a second time ‘shall, upon parole, undergo medroxyprogesterone acetate treatment or its chemical equivalent, in addition to any other punishment prescribed for that offense or any other provision of law’, and submits that ‘[t]his language suggests that the chemical castration is also intended as punishment’.^55^ Both section 645 (a) and (b) refer to MPA or its chemical equivalent being prescribed ‘*in addition to any other punishment*’, which some commentators take to indicate that ALIs are one form of punishment (interpreting the phrase ‘in addition to any *other* punishment’).^56^

Moreover, the original formulation of the provision, in the bill that subsequently became section 645 of the California Penal Code,^57^ *did* contain correctional language. It read ‘[a]ny person guilty of a third conviction of any of the following offenses shall be *punished by chemical castration*’ (emphasis added), which would suggest that it was intended as a form of retribution (although in its enacted form, the phrase *shall be punished* was replaced with *will undergo*).^58^

### E. Conclusions Regarding California

In summary, a number of features of the way in which ALIs are provided under section 645 indicate that they are not intended solely for medical purposes. Offenders are *sentenced* to undergo ALIs by criminal courts, on the basis of the type of offence they have committed, rather than being ordered to undergo ALIs on the basis of an assessment of clinical need. ALIs are administered under the direction of correctional authorities, and while it is within the court's discretion to decide whether to order first time offenders to undergo ALIs, they are mandatory upon commission of a second offence to which section 645 applies. The criteria according to which correctional authorities determine when the administration of ALIs should cease are not specified in the statute, but the fact that there is no requirement that medically trained staff should be involved or consulted in decisions about whether particular offenders should undergo ALIs, either at sentencing, while the intervention is ongoing, or when decisions about whether it should cease are made, suggests that the criteria used are not medical. These considerations all indicate that ALIs provided in accordance with section 645 are intended for correctional and not medical purposes.^59^ The failure to screen for suitability and disregard for empirical evidence regarding the effectiveness of ALIs in preventing recidivism might further indicate that they are intended to realise the objectives of retribution and general deterrence, rather than anti-recidivism.

## IV. ENGLAND AND WALES

This section aims to identify the purposes for which ALIs are used in England and Wales. Our general conclusions are that the approach to the use of ALIs in sex offenders in this jurisdiction is heterogenous, varying widely with the choice of legal regime under which the ALI is provided. When administered under mental health legislation—which is the most common route in England and Wales—ALIs appear to be provided for a mixture of medical and correctional purposes.^60^

### A. Institutional Setting

In England and Wales, ALIs could be administered to sex offenders currently serving sentences in prison, under mental health legislation, or through a mixture of the two (under so-called ‘hybrid orders’ or through transferrals between the two settings). There are currently studies underway within parts of the prison service through which offenders who consent to participation and meet the eligibility criteria might gain access to ALIs.^61^ However, ALIs remain much more likely to be provided to offenders who are detained under mental health legislation, and we will therefore focus on this regime here.

Under the mental health regime, sex offenders will not be *sentenced* to undergoing ALIs. Rather, they will be sentenced either to prison or detention under the Mental Health Act 1983 (MHA 1983).^62^ Once an offender is detained under the MHA 1983, ALIs could be provided to him without his consent,^63^ whether or not he has decisional capacity,^64^ under the direction of the responsible clinician.^65^

Offenders who meet the MHA 1983 admission criteria (detailed below) can be sentenced to detention and care under the Act by a criminal court, but the detention and any interventions provided thereunder are administered by healthcare institutions, sometimes under the direction of the Department of Health, and sometimes under the direction of the Ministry of Justice, or a combination of the two, depending on the type of sentence (see *Conditions of Termination* below). The institutional context within which ALIs are provided under mental health legislation in England and Wales thus does not clearly indicate that they are used either for solely medical or solely correctional purposes. It is perhaps most consistent with their having mixed objectives.

### B. Conditions of Provision

Decisions regarding the imposition of ALIs on particular offenders are taken by the responsible clinician, once a decision to order detention under mental health legislation has been taken by the court. Offenders are sentenced to detention and treatment under mental health legislation through so-called hospital orders, which are issued by criminal courts under s. 37 of MHA 1983 on behalf of individuals who have been convicted of offences punishable by imprisonment, and who meet the admission criteria under section 3(2) of the MHA 1983, which require that the offender:

- … is suffering from mental disorder of a nature or degree which makes it appropriate for him to receive medical treatment in a hospital; and
- it is necessary for the health or safety of the patient or for the protection of other persons that he should receive such treatment and it cannot be provided unless he is detained under this section; and
- appropriate medical treatment is available for him.^66^

These conditions are mostly suggestive of a medical purpose.

However, there are also some indications of a public protection aim. The 2007 amendments to the MHA 1983 incorporated a definition of mental disorder as ‘any disorder or disability of the mind’,^67^ which is broader than the definition contained in the 1983 Act,^68^ and, as Phil Fennell points out, clearly includes paraphilia, personality disorder, and learning disability, which are the forms of mental disorder associated with sex offending,^69^ and therefore renders many sex offenders detainable under the Act.^70^ Indeed, the MHA 2007 Explanatory Notes make it clear that the 2007 amendments to the 1983 Act were intended to bring sexual deviancy of the kind associated with a high risk of sexual offending within the scope of the 1983 Act,^71^ thereby facilitating detention and treatment of both offenders and non-offender patients on the basis of sexual deviance and risk.^72^ Thus, while the requirement that the offender suffers from mental disorder indicates a medical objective, the Act defines mental disorder in a way that appears intended to enable the detention of high-risk offenders.

The grounds for admission under section 3(2)(c) also indicate that detention can serve mixed aims. The requirement under section 3(2)(c) that ‘it is necessary for the health or safety of the patient’ is clearly suggestive of a medical objective. However, section 3(2)(c) also allows for the admission of an offender where it ‘is necessary … for the protection of other persons that he should receive such treatment’.^73^ Where sex offenders are treated under this provision, the aim is arguably to diminish the likelihood of re-offending, with the presence of a mental disorder, and the requirement under section 3(2)(d) that appropriate treatment is available, being relevant primarily as markers of whether treatment is likely to reduce this risk. This, on our model, is a correctional aim.

A further indication that the intent of detention is not purely therapeutic is that a treatment does not necessarily have to be likely to be effective in the particular case in order for the requirement that appropriate medical treatment is available to be met. The requirement that appropriate medical treatment is ‘available’ is satisfied ‘as long as it continues to be clinically appropriate to offer [certain interventions that are available] and they *would* be provided if the patient agreed to engage’.^74^ Thus, while the requirement that appropriate medical treatment is available on the face of it clearly indicates a medical objective, it allows for indeterminate detention also when the ‘care’ will consist in detention only, which may allow for detention to be used also for the prevention of recidivism. Indeed, it has been suggested that the replacement of the ‘treatability’ requirement (in the 1983 Act) with the requirement that appropriate medical treatment is available (through the 2007 amendments) was intended to ‘remove ground for argument about the efficacy or likely efficacy of a treatment, which can be used to prevent detention of people who present a risk to themselves or others’, that is, at least in part in order to facilitate detention for public protection purposes.^75^ Of course, it could be that in such cases the *detention* might be intended partly for the prevention of offending, but that any *interventions* (including ALIs), when provided, are always provided with therapeutic intent. However, it may be legitimate to make inferences about the intended purposes of interventions provided to offenders while detained from the intended purposes of the detention itself, and therefore, if detention were motivated partly by public protection, this at least raises the possibility that any interventions provided to offenders while detained, including ALIs, might be provided partly for the purposes of crime prevention.

Furthermore, there may be some more direct evidence that interventions provided to detained offenders serve a correctional (public protection) purpose, at least in part. While interventions administered to detained offenders must be treatment for mental disorder, that is, their purpose must be ‘to alleviate, or prevent a worsening of, the disorder or one or more of its symptoms or manifestations’,^76^ the courts have accepted that a number of antisocial behaviours, including criminal behaviour such as sex offending, may be manifestations of mental disorder.^77^ ALIs have therefore been accepted as treatment for mental disorder.^78^ Thus, while the requirement that any interventions administered to detained offenders must be treatment for mental disorder may seem to indicate a medical aim, the acceptance of criminal offending behaviours as manifestations of mental disorder arguably suggests that there is also a correctional (public protection) purpose.

### C. Conditions of Termination

In respect of our fourth dimension, conditions of termination, the MHA 1983 offers a range of options for potentially indeterminate detention.

*Hospital orders*, which are imposed by criminal courts, allow for offenders who have been convicted of an imprisonable offence to be detained and treated in hospital until they no longer suffer from a mental disorder that warrants treatment under the MHA 1983.^79^ The absence of a legal time limit and the fact that the length of detention is a reflection of offenders’ treatment needs, suggest a medical objective.

However, the situation is different for *hospital orders with restrictions* (‘*restriction orders*’)*.* These allow criminal courts to impose restrictions relating to offenders’ release, where a hospital order has been made, in situations where this is needed to protect the public from serious harm.^80^ Sex offences will normally justify a restriction order.^81^ The length of the period during which these restrictions apply is determined on the basis of risk to the public, rather than (solely) offenders’ treatment needs, suggesting the aim of detention under these orders is at least in part to reduce the detainee's risk of offending, presumably via either rehabilitation or incapacitation. Moreover, the authority to make decisions to discharge or permit leave for offenders subject to restriction orders lies with the Secretary of State for Justice,^82^ who thereby holds a veto power that limits clinical discretion in respect of these offenders,^83^ again suggesting a correctional aim.

Another type of disposal that seems to reflect a mixture of medical and correctional objectives is ‘*hybrid’* or ‘*Section 45A orders’,* which can be made by courts under section 45A of the MHA 1983. These orders allow for the combination of a hospital direction with a prison sentence^84^ and require a time-limited minimum tariff of detention be served. Offenders whose mental disorder is successfully treated prior to the expiry of their prison sentence will therefore be transferred to prison to serve the remainder of their term. Offenders subject to section 45A orders are initially directed to hospital but have the legal status of prisoners, as opposed to patients.^85^

While *ALIs* may not be provided directly for correctional (public protection) purposes, *detention* under the MHA 1983 can be prolonged for public protection purposes both under restriction orders and section 45A orders, provided that the offender is still suffering from a mental disorder that warrants treatment in hospital, and he is detained, interventions such as ALIs can be provided to him.^86^ In respect of conditions of termination, it thus seems that detention and treatment under mental health legislation may be used for a mixture of medical objectives and for protecting the public via rehabilitation and/or incapacitation.

### D. Language

The language used in the MHA 1983 reflects the mixture of medical and rehabilitative/incapacitative aims noted above. There is a complete absence of language associated with retribution and deterrence, such as references to ‘sanctions’ or ‘punishment’. Measures or interventions provided for under the Act are most commonly referred to as ‘treatment’ or ‘medical treatment’, defined as interventions ‘the purpose of which is to alleviate, or prevent the worsening of, the disorder or one or more symptoms or manifestations’.^87^ The Act also contains words and phrases such as ‘alleviate’ or ‘prevent the deterioration of [the patient's] condition’,^88^ and makes reference to alleviation of ‘serious suffering’ in the patient, and to the patient's ‘needs’, all of which are suggestive of a medical objective.^89^

There are also, however, numerous references to public protection. For example, the criteria for detention under the MHA 1983 refer to it being ‘necessary … for the protection of other persons’.^90^ Moreover, there are references to ‘the protection of others’,^91^ ‘the protection of other persons’,^92^ ‘the protection of the public from serious harm’,^93^ and to interventions preventing the patient from ‘behaving violently or being a danger to himself or others’,^94^ elsewhere in the Act.

The Act also contains numerous references to the measures being necessary for the health or safety of the patient, however,^95^ and often the same provision refers to the measure *either* being necessary for the protection of others *or* being necessary for the health or safety of the patient himself.^96^ The language used in the MHA 1983 thus seems to reflect the mixed medical and correctional objectives that we have identified in respect of institutional setting, conditions of provision, and conditions of termination in the previous sections.

### E. Conclusions Regarding England and Wales

In summary, ALIs provided under the mental health regime in England and Wales seem to combine medical and correctional objectives. While a number of features—for example, the requirement that the offender suffers from mental disorder, and the fact that ALIs are administered under the direction of the responsible clinician—indicate a medical objective, there are also a number of features that indicate that detention and treatment under MHA 1983 may (also) serve a public protection aim. For example, offenders may be detained on grounds of the need to protect third parties, and in respect of some forms of disposals to which an offender can be sentenced (such as ‘restriction’ and ‘section 45A orders’), the authority to grant leave or release lies with the Secretary of State for Justice, rather than responsible clinicians. Offending behaviour has been accepted as a ‘manifestation of mental disorder’, and interventions that target such behaviour is therefore ‘treatment for mental disorder’, and the 2007 amendments of the 1983 Act made it considerably easier to detain offenders on grounds of risk. The provision of ALIs under mental health law in England and Wales may therefore be most aptly described as serving mixed—medical and correctional—purposes.

## V. CONCLUSIONS AND IMPLICATIONS

Existing debate regarding the use of ALIs in sex offenders has tended to assume that valid consent must be obtained before these interventions may permissibly be administered. This suggests that the interventions have been conceived of as serving medical objectives, and not correctional ones, for if their purpose were partly correctional, there would at least be an open question whether consent is required. However, in this article, we have argued that, in some cases, ALIs are administered for correctional purposes, or to realise a mixture of medical and correctional objectives.

In England and Wales, ALIs are typically provided under mental health legislation, where they are described mostly using therapeutic language, and where they are not time-limited. In respect of conditions of provision, only offenders who qualify for a diagnosis of mental disorder are detainable, and decisions about which interventions, including ALIs, ought to be provided to them are made by healthcare professionals. In these respects, ALIs seem to serve medical objectives. However, from the point of view of institutional setting and conditions of termination, things are more complicated. Though ALIs are administered by clinicians in hospital settings, there is, in some cases, a background role for the institutions of criminal justice. For example, the offender may have been involuntarily committed to hospital as part of a criminal sentence, and he may subject to a ‘restriction order’, by which the Secretary of State for Justice holds the power to restrict clinical discretion in respect of decisions to grant the offender leave or release.

Similarly, though the requirement that the offender has a mental disorder and that the ALI is the treatment for that disorder suggests that the *aim* of the ALI is partly therapeutic, the aim of protecting the public through incapacitation and/or rehabilitation also appears to play a role, and indeed is captured in mental health legislation. For example, offenders can be detained under the MHA 1983 (and during detention receive interventions without their consent) either on the basis of this being ‘necessary for the health or safety of the patient’, or on grounds of it being ‘necessary … for the protection of other persons that he should receive such treatment’.^97^ These latter aims are more typically associated with correctional interventions than medical treatments. In some respects then, the use of ALIs under the mental health regime in England and Wales possesses important features of both medical treatments and correctional interventions.

In California, ALIs are arguably used purely, or at least primarily, for correctional objectives. There, ALIs may be imposed as part of criminal sentences, without the involvement of medical professionals or healthcare institutions, and with little regard as to whether they are likely to be effective at mitigating any mental disorder. Though ALIs are sometimes (though not consistently) described using therapeutic language, and their use is, like paradigmatic medical treatments, time-unlimited, it remains plausible to hold that, in California, the main purpose of ALIs is to achieve correctional aims, and more specifically, deterrence and retribution.

The moral implications of our argument are not straightforward. Our argument raises the question whether consent is required for the permissible imposition of ALIs. This is because, if ALIs are intended partly for correctional purposes, it is plausible to think that they should be assessed against the ethical standards of criminal justice,^98^ and those standards are permissive of nonconsensual interventions.

However, though our argument raises this question, it does not answer it. There remains scope to argue that consent *is* required. First, one might argue that, in cases where an ALI serves both correctional and medical purposes, we should ensure that ALIs meet the ethical standards of *both* medicine and criminal justice, and the standards of medicine plausibly include a requirement for valid consent. In that case, the consent requirement could be rejected only in cases where an ALI is intended *wholly* for correctional purposes. Second, one might argue that what ethical standards are applicable to the assessment of ALIs depends not on the *aims* for which they are imposed, but on the nature of the *means* that they employ, and since ALIs employ characteristically medical means—viz., the administration of pharmaceuticals—it might be argued that the relevant ethical standards are those of medicine. Third, one might simply maintain that, where ALIs are being used partly for correctional objectives, they are for that reason being used impermissibly. Thus, the appropriate response is not to change the ethical standards that we used to assess these interventions, but to change the purposes for which they are being used. This view could perhaps be supported by arguing that pharmaceutical interventions are somehow inimical to or inappropriate for the realisation of correctional aims. Finally, it might be argued that ALIs are part of a special subset of correctional interventions that cannot be permissibly administered without consent, for example, because they fall into some special category of ‘extraordinary’ punishments or extraordinary non-punishment correctional interventions.

Regardless, however, a significant change to the existing discussion will be required. Existing discussion has accepted the consent requirement without argument, but if the purposes of ALIs are partly or wholly correctional, this assumption is illicit, for the vast majority of other interventions with a correctional purpose seem to fall under no such requirement. An argument for accepting a consent requirement in relation to ALIs would need to be offered. This argument could perhaps follow one of the four strategies that we have just outlined.

Our argument may also have implications that extend beyond the requirement for consent. Suppose that we were to conclude that in some cases we should indeed assess ALIs against the ethical standards of criminal justice, not those of clinical medicine. Though this might allow considerations of consent to be set aside, it would raise new questions regarding the justification of ALIs. What, precisely, should the aims of criminal justice be, and are ALIs effective means to the realisation of these aims? To what extent should medical professionals become involved in realising the objectives of criminal justice? And, how do ALIs compare, morally, to alternative, more traditional correctional interventions? These are questions that have not figured prominently in debate over the use of ALIs in sex offenders, but to the extent that ALIs ought to be assessed against the standards of criminal justice ethics, neglect of these questions may be difficult to justify.

*Conflict of interest statement*. None declared.

## Footnotes

1 The agents most commonly used for this purpose are medroxyprogesterone acetate, cyproterone acetate, and triptorelin, a gonadotrophin-releasing hormone agonist.

2 For a review of current pharmacological interventions used to lower sex drive in sex offenders, see F Thibaut, F De La Barra, H Gordon, P Cosyns, JMW Bradford, and WFSBP Task Force on Sexual Disorders, ‘The World Federation of Societies of Biological Psychiatry (WFSBP) Guidelines for the Biological Treatment of Paraphilias’ (2010) 11 World J Biol Psychiatry 604.

3 The discussion will be limited to male sex offenders.

4 For the strongest evidence from a single study, see BM Maletzky, A Tolan, and B McFarland, ‘The Oregon Depo-Provera Program: A Five-Year Follow-Up’ [2006] 18 Sex Abuse 303. For a review, see Thibaut et al (n 2).

5 See, eg, DK Ho and CC Ross, ‘Cognitive Behaviour Therapy for Sex Offenders. Too Good to Be True?’ (2012) 22 Crim Behav Ment Health 1.

6 In France, eg, Yves Nicolin of the centre-right Union pour un Mouvement Populaire (UMP) party brought a bill before the Assemblée Nationale in 2009 proposing mandatory ALIs for offenders convicted of rape against victims under 15 years of age, see Assemblée Nationale, N° 2030—Proposition de loi de M. Yves Nicolin visant à rendre visant à rendre obligatoire et permanente la castration chimique pour les coupables d'agressions sexuelles sur mineur de quinze ans, <http://www.assemblee-nationale.fr/13/propositions/pion2030.asp>, accessed 12 December 2015. Two years earlier, Bernard Debré, also of the UMP, brought a bill before the Assemblée Nationale proposing to give criminal courts the power to impose mandatory ALIs on offenders convicted of sexual assault, see Assemblée Nationale, N° 203, Proposition de loi de M. Bernard Debré relative à la castration chimique des criminels sexuels, <http://www.assemblee-nationale.fr/13/propositions/pion0203.asp> accessed 12 December 2015. See also S Gros de Larquier, ‘Interview, Un député UMP veut ‘imposer la castration chimique aux violeurs’’ *Le Point* (Actualités, Politique, 2 October 2009) <http://www.lepoint.fr/actualites-politique/2009-10-02/interview-un-depute-ump-veut-imposer-la-castration-chimique-aux/917/0/382406> accessed 12 December 2015 and S De Larquier, ‘Bernard Debré (UMP): ‘Il faut développer la castration chimique, car il en va de la sécurité de la société’’ *Le Point* (Actualités, Societé, 17 November 2009) <http://www.lepoint.fr/actualites-societe/2009-11-17/bernard-debre-ump-il-faut-developper-la-castration-chimique-car/920/0/395729> accessed 12 December 2015. In Sweden, members of the Christian Democratic Party have previously made such calls, see O Billger, ‘Kd vill kastrera våldtäktsmän’ *Svenska Dagbladet* (Nyheter, Inrikes, 11 September 2002) <http://www.svd.se/nyheter/inrikes/kd-vill-kastrera-valdtaktsman_64221.svd> accessed 12 December 2015.

7 For example Poland, see BBC News, ‘Polish president signs chemical castration law’ (27 November 2009) <http://news.bbc.co.uk/2/hi/europe/8383698.stm> accessed 12 December 2015 and BBC News, ‘Poland castration law takes effect’ (8 June 2010) <http://www.bbc.co.uk/news/10269055> accessed 12 December 2015, and Moldova, see C Rusnac, ‘Moldova Chemical Castration for Convicted Pedophiles Legalized’ *Huffington Post* (The World Post, 6 March 2012) <http://www.huffingtonpost.com/2012/03/06/moldova-chemical-castration_n_1323382.html> accessed 12 December 2015.

8 See for objections about such uses of ALIs, W Green, ‘Depo-Provera, Castration, and the Probation of Rape Offenders: Statutory and Constitutional Issues’ (1986) 12 U Dayton L Rev 1; KA Vanderzyl, ‘Castration as an Alternative to Incarceration: An Impotent Approach to the Punishment of Sex Offenders’ (1994) 15 N Ill U L Rev 107; CL Scott and T Holmberg, ‘Castration of Sex Offenders: Prisoners’ Rights Versus Public Safety’ (2003) 31 J Am Acad Psychiatry Law 502.

9 See, eg, AJ Bond, ‘Antidepressant Treatments and Human Aggression’ (2005) 526 Eur J Pharmacol 218; RM1 Nevels, EE Dehon, K Alexander, and Gontkovsky ST, ‘Psychopharmacology of Aggression in Children and Adolescents with Primary Neuropsychiatric Disorders: A Review of Current and Potentially Promising Treatment Options’ (2010) 18 Exp Clin Psychopharmacol 184.

10 See, eg, SJ Donovan, JW Stewart, EV Nunes, FM Quitkin, M Parides, W Daniel, E Susser, and DF Klein, ‘Divalproex Treatment for Youth with Explosive Temper and Mood Lability: A Double-Blind, Placebo-Controlled Crossover Design’ (2000) 157 Am J Psychiatry 818; LA Khanzode, K Saxena, H Kraemer, K Chang, and H Steiner, ‘Efficacy Profiles of Psychopharmacology: Divalproex Sodium in Conduct Disorder’ (2006) 37 Child Psychiatry Hum Dev 55.

11 T Douglas, ‘Criminal Rehabilitation Through Medical Intervention: Moral Liability and the Right to Bodily Integrity’ (2014) 18 J Ethics 101.

12 See, eg, C Rosati, ‘A Study of Internal Punishment’ [1994] Wis L Rev 123; L Bomann-Larsen, ‘Voluntary Rehabilitation? On Neurotechnological Behavioural Treatment, Valid Consent and (In)appropriate Offers’ (2013) 6 Neuroethics 65; J Ryberg, ‘Punishment, Pharmacological Treatment, and Early Release’ (2012) 26 Int J Appl Philos 231; E Shaw, ‘Direct Brain Interventions and Responsibility Enhancement’ (2014) 8 Criminal Law & Philosophy 1; JC Bublitz and R Merkel, ‘Crimes Against Minds: On Mental Manipulations, Harms and a Human Right to Mental Self-Determination’ (2014) 8 Criminal Law & Philosophy 51; Douglas (n 11).

13 Vanderzyl (n 8) 140.

14 Green (n 8), 16 and 17.

15 See, eg, Rosati (n 12); J Ryberg and TS Petersen, ‘Neurotechnological Behavioural Treatment of Criminal Offenders—A Comment on Bomann-Larsen’ (2013) 6 Neuroethics 79. This is also the legal position in England and Wales: in *Freeman v Home Office (No. 2),* the Court of Appeal accepted that ‘[t]he right approach … is to say that where, in a prison setting, a doctor has the power to influence a prisoner's situation and prospects a court must be alive to the risk that what may appear, on the face of it, to be a real consent is not in fact so’, but held that ‘[e]ssentially … the matter is one of fact’, and that ‘[t]he sole question is therefore whether, on the evidence, there was a real consent’: *Freeman v Home Office (No. 2)* [1984] QB 524 (CA) 542, 543, and 557. Similarly, in the leading case on voluntariness and undue influence, Staughton LJ noted that ‘every decision is made as a result of some influence: a patient's decision to consent to an operation will normally be influenced by the surgeon's advice as to what will happen if the operation does not take place’: *Re T (Adult: Refusal of Treatment)* [1993] Fam 95 (CA) 121. The legal voluntariness test is whether external pressure is such as to overbear the will of the patient and therefore undermines the validity of his consent. This is a question of fact to be established on a case-by-case basis, taking into account (i) the strength of will of the patient and (ii) the relationship between the persuader and the patient: *Re T* ibid 113 (Lord Donaldson).

16 For exceptions to this generalisation, see T Douglas, P Bonte, F Focquaert, K Devolder, and S Sterckx, ‘Coercion, Incarceration, and Chemical Castration: An Argument From Autonomy’ (2013) 10 J Bioeth Inq 393; Douglas (n 11); J Ryberg, ‘Is Coercive Treatment of Offenders Morally Acceptable? On the Deficiency of the Debate’ (2015) 9 Criminal Law & Philosophy 619.

17 For a similar analysis of existing debate, see Ryberg (n 16). Though Ryberg does not address the question whether ALIs ought to be descriptively characterised as medical treatments or correctional interventions, he does note that existing debate has tended to employ ethical principles from medical ethics, rather than penal theory. See Section 5 below for more on Ryberg's argument.

18 An alternative explanation would be that those who have accepted the consent requirement have assumed that ALIs *ought* to be imposed only for medical purposes, though they are not currently (we return to this view below). However, if this were the assumption, it would be difficult to explain why the objection to ALIs has been presented as one focused on the lack of consent, not on the inappropriate purposes for which ALIs are imposed.

19 One such exception in respect of infectious disease is discussed in M Brazier and J Harris, ‘Public Health and Private Lives’ (1996) 4 Med L Rev 171. It is worth noting that for the purposes of the law, consent is rarely an individually sufficient condition for the lawfulness of medical intervention, eg in England and Wales, a public interest justification is also needed as a jointly sufficient condition for the lawfulness of interventions that involve injury above a certain threshold (‘actual bodily harm’ (*R v Brown (Anthony Joseph)* [1994] 1 AC 212 (HL)), and physicians are never under a duty to provide interventions that are not, in their professional judgement, in the patient's best interests (*R (on the application of Burke) v General Medical Council* [2005] EWCA Civ 1003).

20 Ryberg (n 16).

21 It is worth noting, however, that some types of sentences, although strictly speaking time limited, will extend over an offender's entire life span, eg this is the case for mandatory life sentences with a whole life tariff and sentences where multiple consecutive terms are given, which is a common sentencing practice in the United States.

22 Although consent requirements under the respective legal regimes may provide a clear indication in respect of whether ALIs are intended for medical or correctional purposes, we will not discuss this issue, since it would risk begging the question.

23 There are also other regimes under which ALIs could be administered in California, eg offenders could be detained under mental health legislation, and potentially provided with ALIs during the course of such detention. Offenders could also be civilly committed under so-called sexually violent predator laws and could receive ALIs while detained, but commitment under such laws tends only to take place once the offender's prison sentence comes to an end, to allow for the continued incarceration of offenders who suffer from a mental disorder or abnormality that is likely to predispose them to reoffending: *Kansas v Hendricks* 521 US 346 (1997); M Cohen and EL Jeglic, ‘Sex Offender Legislation in the United States: What Do We Know?’ (2007) 51 Int'l J. Offend Therapy & Comp. Criminology 369. However, since California has a statute that explicitly provides for the use of ALIs, we have chosen to focus on this regime.

24 Cal Pen Code s 645(a) and (b).

25 RA Lombardo, ‘California's Unconstitutional Punishment for Heinous Crimes: Chemical Castration of Sex Offenders’ (1997) 65 Fordham L Rev 2611, 2620.

26 The relevant offences are ‘sodomy’, ‘forcible sodomy’, ‘aiding and abetting forcible sodomy’ (s 286), ‘a forcible lewd or lascivious act’, ‘oral copulation’, ‘forcible oral copulation’, ‘aiding and abetting forcible oral copulation of a person’ (s 288), and ‘forcible penetration with a foreign object’ (s 289), all where the victim is under the age of 13 years.

27 Cal Pen Code, s 645(a).

28 Ibid, s 645(b).

29 Ibid, s 645(d).

30 This has been noted by several commentators, see, eg, K-F Brody, ‘A Constitutional Analysis of California's Chemical Castration Statute’ (1997–1998) 7 Temp. Pol. & Civ. Rts. L. Rev. 141, 145; MV Daley, ‘A Flawed Solution to the Sex Offender Situation in the United States. The Legality of Chemical Castration for Sex Offenders’ (2008) 5 Ind. Health Law Rev. 87, 107; A Moog, ‘California Penal Code Section 645: Legislators Practice Medicine on Child Molesters’ (1998–1999) 15 J Contemp Health L & Pol'y 711, 715.

31 See, eg, KW Druhm, ‘A Welcome Return to Draconia: California Penal Law §645, the Castration of Sex Offenders and the Constitution’ (1997–1998) 61 Alb L Rev 285, 304; Brody (n 30).

32 Moog (n 30).

33 See, eg, L Beckman, ‘Chemical Castration: Constitutional Issues of Due Process, Equal Protection, and Cruel and Unusual Punishment’ (1997–1998) 100 W Va L Rev 853, 859. Indeed, Del Busto and Harlow note that there are some data indicating that many, or most, sex offenders whose offences involve child molestation do not suffer from paraphilias, see E Del Busto and MC Harlow, ‘American Sexual Offender Castration Treatment and Legislation’ in DP Boer, R Eher, LA Craig, MH Miner, and F Pfäfflin (eds), *International Perspectives on the Assessment and Treatment of Sexual Offenders: Theory, Practice, and Research* (John Wiley & Sons Ltd. 2011), 547. See also R Hanson and K Morton-Bourgon, ‘The Characteristics of Persistent Sexual Offenders: A Meta-analysis of Recidivism Studies’ (2005) 73 J Consult Clin Psych 1154.

34 EA Fitzgerald, ‘Chemical Castration: MPA Treatment of the Sexual Offender ’ (1990) 18 Am J Crim L 1, 5. This is noted by a number of other commentators too, eg Beckman (n 33) and Moog (n 30).

35 See, eg, KJ Rebish, ‘Nipping the Problem in the Bud: The Constitutionality of California's Castration Law’ (1998) 14 NY L Sch J Hum Rts 507, 519; Moog (n 30); PJ Henderson, ‘Section 645 of the California Penal Code: California's "Chemical Castration Law"—A Panacea or Cruel and Unusual Punishment?’ 32 U S F L Rev 653, 653.

36 Moog (n 30).

37 Cal Pen Code, s 645(d).

38 Druhm (n 31).

39 Lombardo (n 25) 2619.

40 Thibaut et al (n 2). This has been noted by numerous commentators, see eg Rebish (n 35); C Gilchrist, ‘An Examination of the Effectiveness of California's Chemical Castration Bill in Preventing Sex Offenders from Reoffending’ (1998–1999) 7 South Calif Interdiscip Law J 181, 184; PJ Gimino, ‘Mandatory Chemical Castration for Perpetrators of Sex Offences Against Children: Following California's Lead’ (1997) 25 Pepp L Rev 67, 97, and was also noted in the case of *Michigan v Gauntlet* 352 NW2d 310, 314 (Mich Ct App 1984), which considered the lawfulness of judicially imposed ALIs as a condition of probation.

41 Fitzgerald (n 34) 3. See, eg, Ga Code Ann (1998), s 16-6-4(d)(2), which requires that ALIs ‘must be coupled with treatment by a qualified mental health professional’.

42 Brody (n 30), 145.

43 Lombardo (n 25), 2619.

44 Ibid.

45 See, eg, Del Busto and Harlow (n 33); R Hanson and M Bussiere, ‘Predicting Relapse: A Meta-analysis of Sexual Offender Recidivism Studies’ (1998) 66 J Consult Clin Psych 348; Hanson and Morton-Bourgon (n 33).

46 Often, interventions will need to be effective as medical treatments in order to work as public protection measures, KL Smith, ‘Making Pedophiles Take Their Medicine. California's Chemical Castration Law’ (1998–1999) 17 Buff Pub Int L J 123, 148; HA Smith, ‘Common Enemy and Political Opportunity Leave Archaically Modern Sentencing Unchecked: The Unconstitutionality of Louisiana's Chemical Castration Statute’ (2013) 59 Loy L Rev 211, 214.

47 Cal Pen Code, s 645(d).

48 Druhm (n 31) 302. This is also noted by, eg, Gilchrist (n 40), 189 and Daley (n 30), 108.

49 Brody (n 30), 164.

50 Since the enactment of section 645, a form of hearing procedure has been established by the Department of Corrections, which is to be used for the purposes of determining whether the continued use of ALI is necessary. There is no indication that decisions are based on offenders’ clinical needs, however, although they do take into account ‘parolee has a medical condition requiring termination of the treatments’, see Moog (n 30), 731.

51 Druhm (n 31) 313.

52 Ibid, 313 and 314.

53 Ibid, 314.

54 Ibid, 314.

55 Ibid, 313.

56 Ibid, 313; Henderson (n 35), 666; and Gilchrist (n 40), 197.

57 Assembly Bill 3339, introduced on 17 April 1996 by Assembly Member Bill Hoge. The bill was signed into law by California's then governor Peter Wilson on 17 September 1996, taking effect for convictions occurring after 1 January 1997. See Moog (n 30), 725. California law provided for ALI use also before the 1997 amendment of section 645, but ‘prior law had left it within the discretion of the judge to offer Depo-Provera as a choice of punishment to a paroled repeat offender’, see Lombardo (n 25), 2611.

58 This has been noted by several commentators: eg Henderson argues that ‘[o]ne can make a strong argument that the drafters of the law intended the administration of MPA to be punishment’, see Henderson (n 35), 666, and Lombardo submits that ‘the statute's legislative history reveals that it was intended to be a punitive measure’, see Lombardo (n 25), 2620. Moog notes that at the time, ‘Assemblyman Hoge stated that the purpose of the bill was to prevent or lessen recidivism’, see Moog (n 30), 726. However, Hoge also made other statements that seem to contradict this, eg he stated that ‘[t]his legislation sends a clear message to child molesters—you are not welcome in California, and if you commit these heinous crimes, you will be dealt with appropriately’, see Rebish (n 35), 508.

59 It might be argued that section 645 is *intended* to achieve a medical purpose, but that it is poorly drafted, thus wrongly giving the impression of a correctional purpose. We acknowledge that this is a possibility. However, it remains reasonable to assume that the conditions and wording used in the enacted legislation has some evidential value in revealing the state's intentions. If this is correct, then the conditions and wording used in section 645 give some evidence for a correctional purpose, though this might well be defeated by countervailing evidence.

60 We argue that ALIs might be offered for correctional purposes, but we acknowledge that it may not be lawful to provide them for purely correctional purposes, since physicians may only lawfully provide interventions that are, in their clinical judgement, in the best interests of the patient (*Burke* (n 19)). In addition, where the interference with the offender's bodily integrity amounted to actual bodily harm, it would only be lawful if it fell within the scope of the ‘medical exception’, that is, the public interest justification for the lawfulness of medical interventions (*Brown* (n 19)). The medical purpose would therefore be the justifying purpose, while the correctional purpose would be secondary. It is also acknowledged that human rights law—and in particular Articles 3 (which protects individuals from inhuman or degrading treatment or punishment) and 8 (which protects individuals’ right to private and family life)—may place further constraints on the kinds of interventions that could be lawfully provided.

61 See eg L Shaw, ‘Sex Offenders in Pilot Drugs Trial’ *BBC News* (11 June 2012) <http://www.bbc.com/news/uk-18402203> accessed 12 December 2015.

62 Mental Health Act 1983 (as amended by Mental Health Act 2007). Diversion into the mental health system rather than a custodial sentence is an option if the defendant meets the MHA admission criteria. However, there is considerable judicial and medical discretion in respect of which route is chosen, and many eligible offenders are not diverted for detention under the MHA: email from Genevra Richardson (9 October 2015) and phone call from Callum Ross (9 October 2015) to the author.

63 Under s 63 of MHA 1983, interventions which are treatment for the mental disorder the offender suffers from may be given to him without his consent (with the exception of interventions covered by sections 57, 58, and 58A, that is, psychosurgery or hormonal ALI implants (s 57), psychiatric medicines administered for periods longer than 3 months (s 58), or electroconvulsive therapy (s 58A), under the direction of the approved clinician in charge of the treatment. ALIs that are psychiatric medicines (and therefore exempted from s 63) may be administered without the offender's consent under section 58, subject to the requirement for a second medical opinion.

64 Mental Capacity Act 2005, ss 2, 3.

65 Sometimes a requirement of a second opinion by another clinician.

66 MHA 1983, s. 3(2).

67 Ibid, s 1.

68 The ‘treatability test’ in the1983 Act stated that an intervention should be ‘likely to alleviate or prevent a deterioration of [the patient's] condition’.

69 P Fennell, ‘Sex Offenders, Consent to Treatment and the Politics of Risk’ in K Harrison and B Rainey (eds), *The Wiley-Blackwell Handbook of Legal and Ethical Aspects of Sex Offender Treatment and Management* (John Wiley & Sons, Ltd. 2013) 41.

70 Ibid. See also Mental Health Act 2007: Explanatory Notes, para. 24.

71 See also Mental Health Act 2007: Explanatory Notes, paras 23 and 24. This is also noted by Fennell (n 69), 41.

72 Fennell (n 69), 39. Prior to the enactment of the 2007 amendments, section 1(3) of the MHA 1983 stated that individuals could not be treated as suffering from a mental disorder for the purposes of treatment under the Act ‘by reason only of promiscuity or other immoral conduct, sexual deviancy or dependence on alcohol or drugs’. The 2007 amendments removed this provision, since the original formulation was thought to constitute an obstacle to the detention of individuals suffering from personality disorders, where these primarily manifest as sexual deviancy.

73 MHA s 3(2)(c).

74 Department of Health, *Code of Practice Mental Health Act 1983* (London: TSO, 2008), para. 6.19.

75 Joint Committee on Human Rights, *Legislative Scrutiny: Mental Health Bill. Fourth Report of Session 2006–07* (HL Paper 40, HC Paper 288, 2007), 45.

76 MHA 1983, s 145(4).

77 Fennell (n 69), 51.

78 *R v Mental Health Act Commission, ex parte W* (1988) 9 BMLR 77 (CA). Here, it was held that interventions that targeted the sexual offending behaviour were ‘inextricably linked with the mental disorder’ and that ALIs therefore were ‘treatment for mental disorder’.

79 MHA 1983, s 37. In practice, these can result in a substantial period of detention, eg the ‘Moors murderer’ Ian Brady has been detained under MHA 1983 in Ashworth Hospital since he was transferred there from prison in 1985, after having been sentenced to life imprisonment in 1966: *R v Collins and another, ex parte Brady* (2000) 58 BMLR 173 (QBD).

80 ‘Serious harm’ need not involve personal injury, nor need there be a risk to the general public: *R v Birch* (1989) 11 Cr App R (S) 202.

81 Fennell (n 69), 45.

82 Ministry of Justice National Offender Management Service, *Guidance for Working with MAPPA and Mentally Disordered Offenders* (2010) 15 <http://www.justice.gov.uk/downloads/offenders/mentally-disordered-offenders/mappa-mental.pdf> accessed 12 December 2015. The same applies to decisions to transfer such offenders between hospitals. In practice, however, decisions are delegated to the Mental Health Casework Section. The Mental Health Casework Section was until 2009 known as the Mental Health Unit.

83 Ibid, para. 2.5. The one exception to this veto power is if the Mental Health Tribunal orders the discharge of the offender unless, they are satisfied that the criteria for detention are met. The Secretary of State holds no power to veto decisions made by the Tribunal.

84 Ministry of Justice, *Mental Health Act 2007: Guidance for the courts on remand and sentencing powers for mentally disordered offenders* (2008) 6 <https://www.justice.gov.uk/downloads/offenders/mentally-disordered-offenders/guidance-for-the-courts-mha.pdf>. This possibility has, however, been underused in the past, see CC Ross, DK Ho, M Veisi, and T Gill, ‘Treating Sexual Offenders: Current Limitations and Effective Solutions’ (unpublished manuscript). Hybrid orders under s. 45A of the MHA 1983 are only available for sentences that are not subject to a term of imprisonment fixed by law, see MHA 1983, s. 45A(1).

85 Fennell (n 69), 47.

86 Offenders can challenge decisions to extend their detention by appealing to the Mental Health Tribunal, who must, if they find that the criteria for continued detention are not met, direct the offender's discharge. The criteria according to which Mental Health Review Tribunals make their decisions are set out in s 72 of MHA 1983. These criteria are the same the s 3 MHA 1983 criteria for detention. Article 5 ECHR also protects the right for offenders to have their detention reviewed at regular intervals. The Law Commission is at the time of writing conducting a consultation on deprivation of liberty safeguards in respect of offenders who lack decision-making capacity, see <http://www.lawcom.gov.uk/project/mental-capacity-and-deprivation-of-liberty/>.

87 MHA 1983, 145(4).

88 See eg MHA 1983, ss 3(2)(b), 16(2), 20(4)(b), 37(2)(a)(i), and 47(1)(b).

89 See, eg, ibid, s 13(2).

90 Ibid, s 3(2)(c).

91 Ibid, s 5(4)(a).

92 See, eg, ibid ss 2(2)(b), 7(2)(b), 17(1), 17(3), 17(4), 20(2)(c), 20(7)(b), 72(1)(a)(ii), 72(1)(b)(ii), 72(4)(b), 134(2), and 136(1).

93 MHA 1983, ss 41(1) and 42(1) (in respect of restriction orders).

94 MHA 1983, s 62(1)(d).

95 Ibid, ss 2(2)(b), 3(2)(c), 5(4)(a), 12(1)(a), 17(4), 20(4)(c), 72(1)(a)(ii), and 72(1)(b)(ii).

96 Ibid, ss 2(2)(b), 17(4), 20(4)(c), 72(1)(a)(ii), and 72(1)(b)(ii).

97 MHA 2007: Explanatory Notes, para. 24.

98 Here, we are buttressing a point made in Ryberg (n 16). Ryberg argues that debate regarding the coercive use of medical interventions in criminal offenders has neglected penal theory and has too quickly assumed that this is a debate that can be resolved using the principles of medical ethics. Our argument might seem to provide strong support to Ryberg's, for if ALIs are (sometimes) intended in part to achieve correctional objectives, then the relevance of penal theory to their justification will be clear.

